# How to Better Motivate Customers to Participate in the Self-Design Process: A Conceptual Model in Underlying Self-Congruence Mechanism

**DOI:** 10.3389/fpsyg.2020.01995

**Published:** 2020-08-06

**Authors:** Baojun Yu, Hangjun Xu, Brooke Emery

**Affiliations:** ^1^Department of Management Science and Engineering, School of Management, Jilin University, Changchun, China; ^2^Department of Marketing, McAfee School of Business, Union University, Jackson, TN, United States

**Keywords:** actual self-congruence, ideal self-congruence, self-design process, willingness to participate, self-concept

## Abstract

The voluntary shift of responsibility from the producer to the consumer is one feature of self-design activities. Past research emphasizes the economic gains of such customer co-creation. However, the psychological mechanism underlying customer co-creation behavior is still not fully understood. Notably, the goal-driven self-congruence nature of customer co-creation is mostly ignored in the co-creation literature. The objective of this research is to firstly develop a conceptual understanding of how co-creation literature can be related to the self-congruence theory. Furthermore, this study also extends the original self-congruence theory by arguing the differential role of actual and ideal self-congruence on the relationship between self-congruence and customers’ willingness to participate in the co-creation process. Two laboratory experiments were conducted to examine whether self-congruence plays a prominent role in motivating customers to participate in the self-design process. Specifically, both the actual self-congruence and ideal self-congruence are positively related to customers’ willingness to participate in the self-design process is hypothesized. Moreover, it is expected that product styles and different consumption situations may strengthen the relationship between actual/ideal self-congruence and customers’ willingness to participate in the self-design process. Theoretical and practical implications are also discussed.

## Introduction

Previous research has defined customer co-creation as “the joint creation of value by the company and the customer; allowing the customer to co-construct the service experience to suit their context” (Prahalad and Ramaswamy, 2004, p. 8). In addition, Grönroos and Voima (2013) rigorously analyzed the differences between value creation and co-creation with a focus on the roles of the customer and the firm. Generally, value creation entails a process that increases and applies the customer’s knowledge and skills to convert the potential value into real value (value-in-use), while co-creation emphasizes the role of the customer evolved from self-service, through the firm-scripted staging of customer experiences, to co-designing and finally co-production of service (Grönroos and Voima, 2013; Prahalad and Ramaswamy, 2004; Vargo and Lusch, 2004)^1^. Given the importance of the customer co-creation, many studies have been conducted on this topic (Bettencourt, 1997; Prahalad and Ramaswamy, 2000, 2004; Vargo and Lusch, 2004; Fang, 2008; Chan et al., 2010; Grönroos and Voima, 2013; Guo et al., 2013; Dong and Sivakumar, 2017). Previous studies on co-creation literature examine customers’ involvement in the product design process, either self-design/customize a product (e.g., LLBean backpack; Moreau and Herd, 2010) or make the resulting product (e.g., making dinner with a boxed meal; Troye and Supphellen, 2012).

One feature of self-design activities is the voluntary responsibility shift from the producer to the consumer (Moreau and Herd, 2010). More and more companies facilitate and maintain their competitive advantage through the competence of customers by shifting them from passive value receivers to active value creators (Prahalad and Ramaswamy, 2000). Products catering to “self-expression for the time-deprived” has created the demand for new product offerings by firms ranging from sports shoes (e.g., Nike ID’s custom design service), to creative construction toys (e.g., Lego) to home improvement (e.g., Lowe’s). For example, Lego provides an online community, ideas.lego.com, to bring together passionate fans and creators from around the world to imagine, create, and evaluate ideas for new Lego sets. Once selected, the customized design becomes a real Lego set that can be purchased around the world with royalties paid to the set’s creator. No wonder recent research has demonstrated that consumers are willing to pay a significant premium for self-design or customized products relative to comparable mass-produced counterparts (Franke and Piller, 2004; Franke et al., 2009; He et al., 2016).

Although most previous research focuses on explaining the reasons why co-creation activities bring positive outcomes to customers or/and companies, such as self-designed products better satisfy consumer needs, consumers have higher purchase intention for those self-designed products, and consumers are more willing to recommend the self-designed product (Moreau and Herd, 2010; Moreau, 2011; He et al., 2016; Hsieh and Chang, 2016; Wiecek et al., 2019), little attention has focused on how to motivate customers to participate in the self-design process. A key concept for investigating this question is the concept of “self-congruence” (Sirgy, 1982; Sirgy and Samli, 1985; Aaker, 1999), which fits the product’s personality or brand with the consumer’s self-value. Previous literature has suggested that self-congruence can enhance affective, attitudinal, and behavioral consumer responses to the brand and outcome (Aaker, 1999). Based on this perspective, this study examines the role self-congruence plays in motivating customers to participate in the self-design process. The better the fit between the product’s personality and the consumer’s self-value, the more likely the customer will participate in the self-design process. Furthermore, this study gains insight into how actual self-product image congruence and ideal self-product image congruence affect the willingness of customers to participate in the self-design process and how it varies across different product styles and different consumption situations.

This study contributes to the self-congruence theory, customer co-creation literature, and practice in the following ways. First, employing the self-congruence approach, this study examines the critical role played by self-congruence in customer co-creation, which has been largely neglected in the co-creation literature. Furthermore, instead of measuring general self-congruence, we take an initial step to create a special approach to manipulate self-congruence into two different perspectives (e.g., actual self-congruence and ideal self-congruence). To the best of our knowledge, this is the first study to manipulate self-congruence, which broadens our understanding of the causal relationship between self-congruence and customer co-creation behavior. Finally, this study also explores the consumption situation and product styles as moderators of the relationship between (actual and ideal) self-congruence and willingness of customer participation in the self-design process.

The remainder of the article is organized as follows. First of all, we outline the literature review of customer co-creation and develop our framework and propositions based on a synthesis of the literature. Subsequently, we present the research design. Finally, the conclusion section summarizes our major expected findings, contributions, and limitations of this study.

## Literature Review and Theoretical Background

### Customer Co-creation Literature

Previous research has defined customer co-creation from different perspectives. Customer co-creation refers to customers’ involvement in company-based tasks that are related to sharing innovation, design, and/or ideas generations (Grewal et al., 2006; Grönroos, 2017). Based on a review of existing customer co-creation literature, we summarized the previous findings in the following section.

#### The Antecedents of Customer Co-creation

Prior research explained why some consumers are more willing and able to engage productively in the value co-creation process than others (Etgar, 2008; Füller, 2010; Dong and Sivakumar, 2017). We classified them into two categories: personal factors and organizational factors.

##### Personal factors

According to self-determination theory, consumers’ motives to participate in the co-creation process can be considered a function of either intrinsic motivation or extrinsic motivation (Ratner et al., 1999; Deci and Ryan, 2000).

In terms of intrinsic motivation, co-creation may generate excitement in consumers and satisfy their variety-seeking needs (Ratner et al., 1999), such as the sense of self-expression and pride (Etgar, 2008), creative achievements (Burroughs and Mick, 2004) and the enjoyment of contribution (Evans and Wolf, 2005; Nambisan and Baron, 2009). Moreover, some consumers may participate in the co-production or service process purely driven by a sense of altruism (Nambisan and Baron, 2009). In sum, the feeling of autonomy, competence, task enjoyment, perceived control and sense of community will promote the co-creation experience, which will drive customers’ interest in future participation (Füller et al., 2011; Guo et al., 2013, 2016; Hsieh and Chang, 2016).

In terms of extrinsic motivation, co-creation may offer consumers’ opportunities to obtain some valuable results, such as monetary benefits or financial compensation (Lusch et al., 1992; Füller, 2010). Song and Adams (1993) concluded that monetary incentives could be a useful motivational tool to encourage customers to participate in the service delivery process. Villarroel Fernandez and Tucci (2010) also found that the desire to earn money appears to be the most likely predictor of consumers’ participation and contribution to co-creation. Boudreau et al. (2011) concluded that “winning cash is the most conspicuous motivation” to participate in TopCoder, an online crowdsourcing community that tests a variety of algorithmic approaches.

##### Organizational factors

Previous studies identified several organizational factors that directly influence customer co-creation, including perceived organizational support, organizational socialization, customer satisfaction, perceived organizational justice/interactional justices, and client–advisor communication.

One major factor that drives customers to participate is perceived organizational support (POS). Eisenberger et al. (1986) proposed that “employees develop global beliefs concerning the extent to which the organization values their contributions and cares about their well-being” to explain the development of employees’ commitment to an organization. According to the notion of a social exchange perspective, greater perceived organizational support will engender a sense of obligation for employees to reciprocate with cooperative behaviors to provide better service to their customers and actively engage them in the value co-creation process. This customer co-creation helps enhance the performance of the organization (Shore and Wayne, 1993; Bettencourt, 1997). In addition, organizational socialization, the process by which an individual adapts to appreciate the values, norms, and certain behavior patterns to an organization (Schein, 1971), can be utilized to provide well-organized customer service with specific behavioral guidelines. The findings from previous studies suggested that customer organizational socialization leads to more accurate role perceptions in consumers and a higher level of willingness to participate in the co-creation process (Kelley et al., 1990). Previous service marketing literature suggests that satisfied customers are likely to provide effective, positive feedback, and information beneficial to the organization (Bhattacharya et al., 1995). Therefore, customer satisfaction is another major factor that influences consumers’ value co-creation behavior. In addition, previous studies also found that both perceived organizational justice/interactional justices (Auh et al., 2007; Yi and Gong, 2008) and client–advisor communication (Auh et al., 2007) are the organizational foundations of customer co-creation.

#### The Outcomes of Customer Co-creation

Regarding customer co-creation outcomes, prior research has explored both the positive and the negative sides of customer co-creation. In the following section, we will discuss the benefits and problems that customer co-creation brings to both firms and consumers.

On the positive side of customer co-creation, previous studies showed that both organizations and customers could benefit from economic values and relational/social values (Chan et al., 2010; Yim et al., 2012; Jaakkola et al., 2015). Economic values refer to the economic benefits of the product or service, whereas relational/social values entail the value derived from emotional or relational bonds between customers and employees (Chan et al., 2010; Yim et al., 2012; Jaakkola et al., 2015). Moreover, marketing practitioners and researchers have increasingly recognized that customer co-creation has positive effects on firm performance by increasing productivity and decreasing costs (Prahalad and Ramaswamy, 2000, 2004). The improvement in firm performance arises from various sources: cost-minimization caused by customers serving as *ad hoc* employees (Lovelock and Young, 1979; Bitner and Brown, 2008), greater repurchases and referrals (Cermak et al., 1994; Shahin and Nikneshan, 2008), higher consumer well-being (Guo et al., 2013; Mende and Van Doorn, 2015), better brand image (Woisetschläger et al., 2008; Shamim et al., 2016), faster response to service failures (Dong and Sivakumar, 2017) and improved service/product development and innovation (Tether and Tajar, 2008; Grissemann and Stokburger-Sauer, 2012). From the customer perspective, customers can accrue economic value through the co-creation process as they benefit from cost reductions and discounts (Prahalad and Ramaswamy, 2004).

Relational/social values derived from the emotional or relational bonds between the customer and the organization may also be a positive consequence for the firm. Co-created products are often shown to improve customer satisfaction (Marzocchi and Zammit, 2006; Chang et al., 2009; Prebensen and Xie, 2017; Auh et al., 2019) and enhance customer loyalty and trust (Auh et al., 2007). A friendly service climate of co-created products/services can increase positive product evaluations (Troye and Supphellen, 2012), positive word of mouth (Woisetschläger et al., 2008), and enriched two-way communication (Claycomb et al., 2001). From the customer perspective, the co-creation process may enhance customers’ skills (Lengnick-Hall, 1996), customer enjoyment (Nambisan and Baron, 2009), and their networking capabilities (Etgar, 2008).

The benefits of customer co-creation for a firm do not come without cost. For example, some uninformed customers may slow down the service process leading them to feel less satisfied with the service (Kelley et al., 1990; Fang, 2008). Some scholars believe that customer co-creation can cause unnecessary uncertainty for service organizations (Fang, 2008), and customers may also become potential competitors to the sellers by gaining the necessary skills to create the offerings independently (Fodness et al., 1993). Research also shows that frustrated customers in the co-creation process may make the employees feel less motivated/productive or even likely to quit (Kelley et al., 1990). Furthermore, the complexity requirements from consumers may increase employees’ perceived workloads and job stress (Hsieh et al., 2004; Chan et al., 2010).

### Consumers’ Motivation to Participate in Co-creation Activities

According to the above review on customer co-creation literature, engaging customers in the value co-creation process has become increasingly important to marketing managers. It is critical to understand how to motivate customers to participate in the production or service delivery process. Bagozzi and Dholakia (1999) contended that most behavior is goal-directed. However, to reach their desired goals, individuals must have some impetus to move forward. This impetus is known as motivation.

Drawing on the rich body of motivation research, Füller (2010) summarized ten different motive categories, such as intrinsic playful task, curiosity, self-efficacy, etc. Fiore et al. (2004) indicated that consumers’ willingness to be involved in co-design is positively related to two motivations: creating a unique product and enjoying the exciting co-design experience. Moreau and Herd (2010) also extend work in social comparison theory by focusing on the motivational and behavioral consequences of the comparison with the self-designed products. In this research, we choose the same phenomenon as our research context and discuss the similarity and difference between customer co-creation activities and self-design activities.

### Similarity and Difference Between Co-creation Activities and Self-Design Activities

Previous research in the co-creation literature has already paid major attention to involving customer engagement in the creation of offerings through ideation, design, development and post-launch process (Etgar, 2008; Vargo and Lusch, 2008; Atakan et al., 2014; Mustak et al., 2016). In recent years, online tools and communities facilitated customer involved their co-creation efforts in the early stage (idea generation and design). In this stage, customer co-creation refers to customers’ involvement in tasks related to innovation, design, and/or production of ideas (Etgar, 2008; Mustak et al., 2016). For example, customers may generate new ideas in companies’ virtual environments or customers could design their own offerings with the help of companies’ self-design tools (Jaakkola et al., 2015). In the product development and commercialization stage, the concept of customer co-creation focuses on the customers’ effort and involvement, both mental and physical, which relate to the production and delivery of a service (Cermak et al., 1994; Mustak et al., 2016). In this stage, the level of customer co-creation can be measured as the extent to which customers will provide or share information, make suggestions, and become involved in decision making during the service co-creation and delivery process (Chan et al., 2010). In the post-launch stage, customer co-creation can be conceptualized as the degree to which customers express their feedbacks by using social media or word of mouth (WOM) to share their positive or negative experiences (Woisetschläger et al., 2008; Heidenreich et al., 2015). In sum, firms might encourage customers to engage in a company’s general offerings (Ramani and Kumar, 2008) or special/unique offerings (Moreau and Herd, 2010; Troye and Supphellen, 2012).

Since self-designed products provide a higher preference fit than standardized products and thus drive customers’ willingness to pay (Franke and Piller, 2004; He et al., 2016), such a special co-creation activity may induce affective reactions and thus increase the value the customer attaches to the product. Previous self-designed research in the co-creation literature also concluded that such premium could be attributed not only to the superior fit with preferences that customized products provide (Franke and Piller, 2004; Franke et al., 2009; He et al., 2016), but also to the sense of accomplishment (the “I Designed it Myself” effect) and ownership (Wiecek et al., 2019) consumers feel when they successfully complete the design process (Moreau and Herd, 2010). Therefore, in this research, we focus on self-design activities as our research context and define the *self-design activities* as a strategy that firms use to give customers a chance to actively engage in the creation of end products, allowing them to select or create the final products for themselves with the company providing production and delivery.

## Theoretical Framework and Propositions Development

**FIGURE 1:**
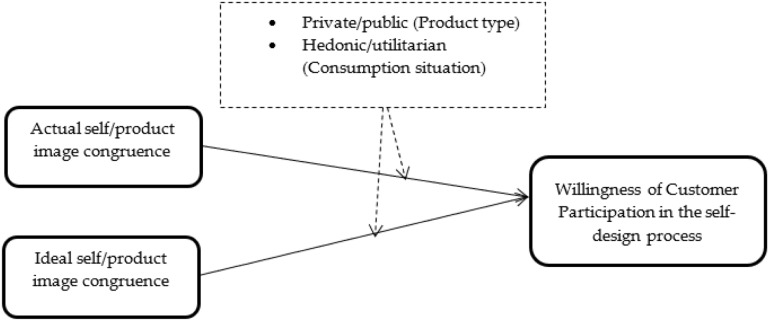
Conceptual framework.

Self-concept is an essential construct in consumer behavior, defined from different perspectives in the existing literature (Sirgy, 1982; Sirgy and Samli, 1985; Sirgy and Su, 2000). In self-congruence theory, the self-concept is defined as the cognitive and affective understanding of who and what we are and can take two forms: the “actual self” and the “ideal self” (Sirgy, 1982; Sirgy and Samli, 1985; Ekinci and Riley, 2003). The actual self is based on who and what I think I am now, whereas the ideal self reflects who and what I would like to be or aspire to become (Sirgy, 1982). Self-product image congruence has been defined as the match between product personality images and customer perceived self-images (Sirgy, 1982; Sirgy and Samli, 1985; Aaker, 1999; Tsai et al., 2015). Actual self-product image congruence reflects the consumer’s perception of the fit between the actual self and the product’s personality, and the ideal self-product image congruence is the perceived fit of the product personality with the consumer’s ideal self (Sirgy, 1982; Sirgy and Samli, 1985). Figure 1 presents our conceptual framework.

### Main Effect of Self-Congruence

According to the cognitive-consistency theory, humans are motivated by inconsistencies and a desire to change them (Festinger, 1957), and consumers are motivated to hold a set of beliefs about themselves (self-concept) that motivate them to act in ways to improve their self-concept. There are two major self-related motives: self-consistency (indicates an individual’s tendency to behave consistently with his/her view of himself/herself) and self-esteem (represents an individual’s tendency to seek self-enhancement) (Sirgy, 1982, 2018; Sirgy and Samli, 1985; Aguirre-Rodriguez et al., 2012; France et al., 2015; Shamim and Ghazali, 2015) may play an important role in this whole psychological process.

In terms of actual self-congruence, people are motivated to verify, validate, and sustain their existing self-concepts (actual-self) (Swann, 1983). Aron and Aron (2005) posited that people possess an inherent motivation to incorporate others (e.g., product) into their conception of self. The more an entity’s product reflects a person’s self-definition, the stronger the customers will want to participate in the self-design process (Wallace et al., 2017; Moliner et al., 2018).

In terms of ideal self-congruence, people may seek information that increases their self-esteem (Ditto and Lopez, 1992). Such self-enhancement motivation drives people to approach their aspirations (i.e., their ideal self) to enhance their self-esteem (Higgins, 1987). Following this logic, a product with a personality that reflects the consumers’ ideal selves can support them in their self-enhancement activities by giving them the feeling of getting closer to their ideal self (Grubb and Grathwohl, 1967). Thus, it is posited that:

P1a: Consumers who perceive their actual-self congruent with the product image will be more willing to participate in the self-design process than those who perceive their actual-self is incongruent with the product image.
P1b: Consumers who perceive their ideal-self congruent with the product image will be more willing to participate in the self-design process than those who perceive their ideal-self is incongruent with the product image.

### The Moderation Role of User Situation

According to previous literature, hedonic consumption is desired for pleasure, fantasy, and fun, whereas utilitarian consumption fulfills basic needs or helps accomplish functional or practical tasks (Strahilevitz and Myers, 1998). Hedonic consumption provides psychological enjoyment rather than being the instrument to achieve aspired ideal self-images. Johar and Sirgy (1991) proposed that the congruency of the ideal self could satisfy the needs for self-esteem. This process transcends the actual-self to attain the perfect self (Sirgy, 1982; Sirgy and Samli, 1985). Customers will have a strong willingness to take part in self-design to achieve their ideal-self, which indirectly meets the need of enhancing self-esteem. Utilitarian consumption mainly focusses on practical and necessary usage, with self-expression consistent with existing self-being the motivation. As a result, the following propositions are offered:

P2a: Consumers are more willing to participate in the self-design process of a utilitarian product than a hedonic product when their actual-self is congruent with the product image.
P2b: Consumers are more willing to participate in the self-design process of a hedonic product than a utilitarian product when their ideal-self is congruent with the product image.

### The Moderation Role of Product Type

According to the previous literature, public products are those that are seen by others when being used, while private products are those products are not seen during the consumption process (Kulviwat et al., 2009). We posited that the relationships between the self-congruence with the product and the willingness to participate in the self-design process are moderated by product type. When a product is consumed primarily in private, the consumer will be less concerned about what others think about the consumption of that specific product. Such consumers will care more about the degree to which the product is satisfactory from the individual’s point of view (actual self-concept). On the other hand, when the product consumption typically takes place in public, the consumer will be more concerned with others’ responses regarding their consumption Thus, the ideal self-concept may be more applicable because people have a basic need to receive approval from society and create positive impressions of themselves in others’ minds. As a result, the following propositions are offered:

P3a: Consumers are more willing to participate in the self-design process of a private product than a public product when their actual-self is congruent with the product image.
P3b: Consumers are more willing to participate in the self-design process of a public product than a private product when their ideal-self is congruent with the product image.

## Method

### Study 1: Research Design

#### Participants

The purpose of Study 1 is to test the main propositions and our moderation effect of the user situation. The study will use a 2 (self-brand congruence: actual vs. ideal) × 2 (user situation: hedonic vs. utilitarian) between-subjects experiment.

#### Procedure and Measures

First, we introduced our participants to Jordan, a college student. Then, we asked that they imagine Jordan visiting a website that sells university-branded products called Udiy (do-it-yourself). Udiy offers products with the university logo that can be personalized (name, major, photo, graduation year, etc.). After viewing the website and the products offered, Jordan decides to customize a watch. The manipulation of the two-user situation control was asked to imagine two different watch styles. The self-brand image congruence manipulation was controlled as follows. *In the actual self-brand image congruence manipulation*, the information states, “Jordan noticed that the face of the watch is customizable with the Ulogo and some messages (name, major, photo, graduation year, etc.). Jordan decides to include the following message that he created: Proud ULife.” *In the ideal self-brand image congruence manipulation*, the information states, “Jordan noticed that the face of the watch is customizable with the Ulogo and some messages (name, major, photo, graduation year, etc.). Jordan decided to include the following message that he created: Fast Hard Finish –U Graduate 2021!”

After reading the assigned scenario, participants reported perceptions of their willingness to participate in the self-design process (with a scale anchored by 1 = “strongly not want to participate,” and 7 = “strongly not want to participate”) (Füller, 2006). Finally, the participants were asked to answer several questions related to manipulation checks and their demographic information.

### Study 2: Research Design

#### Participants

The purpose of Study 2 is to test the moderation effect of product type. The study will use a 2 (self-brand congruence: actual vs. ideal) × 2 (product types: private vs. public) between-subjects experiment.

#### Procedure and Measures

We used the same procedural and self-brand image congruence manipulation as Study 1. In regard to product type manipulation, after the participants looked through the website and the products offered, Jordan decided to customize a handbag (public product) and a toothbrush (private product). Similar to Study 1, after reading the assigned scenario, participants reported their perceptions of willingness to participate in the self-design process. Then, the participants were asked to answer several questions related to manipulation checks and their demographic information.

## General Discussion

As we known, the voluntary shift of responsibility from the producer to the consumer is one feature of self-design activities. However, the psychological mechanism underlying such customer co-creation behavior (e.g., self-design activities) is still not fully understood. To address this above research gap, this study firstly introduces a new angle on self-congruence theory by investigating the motivation of customer participation in the co-creation activities. Specifically, the current conceptual research attempts to examine how self-congruence drives customer co-creation behavior, and furthermore explores how the above relationship is moderated by the consumption situation and product type variables.

### Theoretical Implications

This paper has several implications for marketing research. First, according to self-congruence theory, consumers’ perceptions of consumption objects and their congruence with their own self-concept have been recognized as important determinants of consumer behavior (Sirgy, 1982, 2018; Sirgy and Samli, 1985; Sirgy and Su, 2000; Aguirre-Rodriguez et al., 2012). First, individuals pursuing self-congruence goals put more sustained effort into behaving consistently with his/her view of himself/herself (self-consistency motivation). Second, individuals who attain self-congruence goals reap greater benefits from their tendency to seek self-enhancement (self-esteem motivation). By employing self-congruence theory, we firstly examined the important role played by self-congruence in the customer co-creation field, which extends the co-creation literature by exploring another important psychological mechanism to understand the motivation of customer co-creation behavior.

Furthermore, we also extend the original self-congruence theory by proposing to test the differential role of actual and ideal self-congruence on the relationship between self-congruence and customers’ willingness to participate in the co-creation process. Instead of measuring general self-congruence, we took the initial step to create a special approach to manipulate self-congruence into two different perspectives (e.g., actual self-congruence and ideal self-congruence). To the best of our knowledge, this is the first study to manipulate self-congruence, which broadens the understanding of the causal relationship between self-congruence and customer co-creation behavior. This manipulation approach can be used in future research, both in self-congruence and co-creation research.

### Managerial Implications

This study and its findings have a number of important implications for marketing managers. First, this review shows that customer co-creation can lead to several positive outcomes for customers as well as firms. Customer co-creation can begin as a new tactical element of the marketing mix, but it should evolve into something that is embedded in the strategic fabric of the organization.

In this study, self-congruence is involved as an important mechanism to help understand how to better motivate customers to participate in the self-design process. For example, firms should look beyond their brand image to match the consumer’s self-concept. We also introduced the consumption situation and product styles as the moderator variables to determine which part of the consumer self should be targeted.

### Limitations and Future Research

The findings of this study have several limitations. First, since actual and ideal self-congruence may have a differential role of on the relationship between self-congruence and customers’ willingness to participate in the co-creation process, especially in the service research field. Future research could be conducted to identify self-congruence as an important mechanism in understanding which self-congruence (actual or ideal self-congruence) may better motivate customers to participate in the self-design process. Or, we may test the potential causal relationship between those two self-congruence mechanisms and how those self-congruence variables link to other related outcome variables. Second, although we took an initial step to create a particular approach to manipulate self-congruence into two different perspectives (e.g., actual self-congruence and ideal self-congruence), we may collect data from Amazon Mechanical Turk. And we created our particular scenarios to represent a trade-off between experimental control and external validity. Therefore, we suggest future research should create a real context or field study (such as neuroscience lab) to retest and investigate the role of self-congruence on customer co-creation behavior. Finally, we focused on the moderating effect of the consumption situation and product type on the relationship between self-congruence and customers’ willingness to participate in the self-design process. Some other interesting moderator variables, such as cognitive elaboration, brand personality facet, and impression formation process variables (Aguirre-Rodriguez et al., 2012), should be considered to explore how to better motivate customers to participate in the self-design process.

## Author Contributions

BY and HX: conceptualization. HX: writing – original draft preparation. BE: writing – review and editing. All authors contributed to the article and approved the submitted version.

## Conflict of Interest

The authors declare that the research was conducted in the absence of any commercial or financial relationships that could be construed as a potential conflict of interest.

## Footnotes

1 We thank reviewer to focus on this issue.
